# Epigenetic Control of Osteogenic Lineage Commitment

**DOI:** 10.3389/fcell.2020.611197

**Published:** 2021-01-08

**Authors:** Martin Montecino, Margarita E. Carrasco, Gino Nardocci

**Affiliations:** ^1^Faculty of Medicine and Faculty of Life Sciences, Institute of Biomedical Sciences and FONDAP Center for Genome Regulation, Universidad Andres Bello, Santiago, Chile; ^2^Faculty of Medicine, Universidad de los Andes, Santiago, Chile; ^3^Molecular Biology and Bioinformatic Lab, Program in Molecular Biology and Bioinformatics, Center for Biomedical Research and Innovation (CIIB), Universidad de los Andes, Santiago, Chile

**Keywords:** epigenetic control, bone-related expression, osteoblast differentiation, histone marks, chromatin

## Abstract

Within the eukaryotic nucleus the genomic DNA is organized into chromatin by stably interacting with the histone proteins as well as with several other nuclear components including non-histone proteins and non-coding RNAs. Together these interactions distribute the genetic material into chromatin subdomains which can exhibit higher and lower compaction levels. This organization contributes to differentially control the access to genomic sequences encoding key regulatory genetic information. In this context, epigenetic mechanisms play a critical role in the regulation of gene expression as they modify the degree of chromatin compaction to facilitate both activation and repression of transcription. Among the most studied epigenetic mechanisms we find the methylation of DNA, ATP-dependent chromatin remodeling, and enzyme-mediated deposition and elimination of post-translational modifications at histone and non-histone proteins. In this mini review, we discuss evidence that supports the role of these epigenetic mechanisms during transcriptional control of osteoblast-related genes. Special attention is dedicated to mechanisms of epigenetic control operating at the Runx2 and Sp7 genes coding for the two principal master regulators of the osteogenic lineage during mesenchymal stem cell commitment.

## Introduction

Osteoblast lineage commitment is regulated by a coordinated set of extra cellular stimuli and developmentally-regulated signaling pathways, including those mediated by bone morphogenic proteins (BMPs), Wnt-ligands, steroid hormones, and growth factors, among others (Li et al., 1998; Nishimura et al., 1998; Drissi et al., 2000; Yamaguchi et al., 2000; Zhang et al., 2008). Following activation in pre-osteogenic cells, these signaling pathways modulate the expression and function of osteoblast master transcription factors, which in turn control the expression of downstream bone-phenotypic genes, thus establishing the osteoblastic cell component of the mammalian skeleton (Stein et al., 2004; Long, 2012). Although the precise molecular mechanisms associated with transcriptional control of these osteoblast-related genes are still far from being completely understood, significant progress has been made during the last two decades.

We discuss research demonstrating the role of epigenetic mechanisms in controlling gene transcription during mesenchymal cell commitment to the osteogenic lineage. We first overview key components of epigenetic mechanisms that control gene expression in mammals and then describe how these epigenetic processes contribute to regulate transcription of the Runx2 and Sp7 genes, two critical osteogenic master regulators.

## General Overview of Epigenetic Control in Mammals

Nuclear DNA is organized as chromatin. Nucleosomes are the fundamental units of this chromatin and are composed of an approximately 147 bp DNA segment wrapped around an octamer of histone proteins (two each of histones H2A, H2B, H3, and H4) (Ramakrishnan, 1997; Richmond and Davey, 2003). A portion of these histone proteins (N-terminus tail) extends beyond the limits of the particle core, providing additional surfaces for interaction with nuclear proteins that regulate transcription (Strahl and Allis, 2000; Bannister and Kouzarides, 2011; Voigt et al., 2013). As specific residues (e.g., lysines and arginines) within these histone tails are subject to extensive enzymatic post-translational modifications, the potential for modifying the chemical environment surrounding the chromatin fibers represents a key regulatory component during gene expression control (Bannister and Kouzarides, 2011). The genomic DNA can be also enzymatically modified affecting the degree of compaction of chromatin. Increased condensation reduces the possibility that specific regulatory DNA sequence motifs are recognized by transcription factors that control transcription (Franchini et al., 2012; Bogdanović and Lister, 2017).

Molecular components that regulate chromatin organization and transcription are critical players during cell differentiation. Among them the large group of “histone post-translational modifications” (HPTMs), which are considered a principal “epigenetic” mechanism (Bannister and Kouzarides, 2011; Shilatifard, 2012; Voigt et al., 2013). HPTMs may function as docking sites on the chromatin fiber surface that can be recognized by regulatory proteins (“epigenetic readers”) that contain specific complementary domains (e.g., chromo-domains interact with methylated histone lysine residues) (Voigt et al., 2013; Venkatesh and Workman, 2015). A number of protein complexes that are capable of mediating deposition (“epigenetic writers”) or elimination (“epigenetic erasers”) of HPTMs have been identified in eukaryotic cells. Importantly, most of the core subunits of these complexes are evolutionary conserved (Dimitrova et al., 2015; Piunti and Shilatifard, 2016; Jambhekar et al., 2019), indicating that their functions during gene expression control are also conserved across species.

Genetic studies allowed the identification of the Polycomb Group (PcG) and the Trithorax Group (TrxG) complexes, which mediate inhibition and activation of transcription, respectively, during embryogenesis (Ringrose and Paro, 2007). One signature property of the evolutionary conserved PcG complexes PRC1 and PRC2 (Polycomb Repressive Complex 1 and 2) is to mediate the formation of repressed chromatin. In mammals, PRC2 includes as main subunits Enhancer of Zeste Homolog 2 or 1 (Ezh2/Ezh1), Suppressor of Zeste 12 (Suz12), and Embryonic Ectoderm Development (Eed) (Piunti and Shilatifard, 2016; Yu et al., 2019). Ezh2 (or alternatively Ezh1) is the main catalytic component of PRC2 mediating tri-methylation of the lysine 27 residue of histone H3 (H3K27me3), modification that is associated with transcriptionally silent chromatin (Voigt et al., 2013; Piunti and Shilatifard, 2016).

Several mammalian TrxG complexes, including COMPASS (Complex of Proteins Associated with Set1a/b) and the Mixed Lineage Leukemia (MLL1–5)-containing COMPASS-like complexes have been reported (Shilatifard, 2012). TrxG-mediated activity involves mono-, di-, and tri-methylation of the lysine 4 residue of histone H3 (H3K4me1, H3K4me2, and H3K4me3). H3K4me3 is often found enriched at transcriptionally active chromatin (euchromatin), mainly around the transcription start sites (TSSs) of promoters. Moreover, H3K4me3 can be recognized by the RNA polymerase II complex, hence facilitating transcriptional activity at H3K4me3-marked gene promoters (Vermeulen et al., 2007). Set1-COMPASS and MLL-COMPASS-like complexes include the Wdr5 (WD Repeat Domain 5) subunit which has been shown necessary for assembly, stability and optimal enzymatic activity of these complexes (Shilatifard, 2012). Interestingly, some of these histone-methylating complexes also include enzymatic activities that can remove other histone marks. For instance, MLL3/4-COMPASS-like that also contains the H3K27me3 demethylase Utx/Kdm6a (Piunti and Shilatifard, 2016). Hence, binding of MLL3/4-COMPASS-like to a particular genomic region can also result in reduced H3K27me3, an epigenetic signature associated with decreased transcription (Voigt et al., 2013; Piunti and Shilatifard, 2016).

Set1-COMPASS mediates global genomic deposition of H3K4me3 in most mammalian cells and therefore its function is tightly associated with transcriptional activation of a large number of genes. MLL2-COMPASS-like has been shown responsible for H3K4me3 deposition at promoters in embryonic stem cells (ESCs), contributing to transcriptional upregulation of homeobox genes (Denissov et al., 2014). Interestingly, MLL3/4-COMPASS-like can catalyze the deposition of H3K4me1 at enhancer elements in mammalian cells (Hu et al., 2013; Yan et al., 2018). Recent results from several teams, however, also show that MLL3/4 complexes can mediate the maintenance of the H3K4me1 mark at proximal promoter regions of repressed, but poised for expression, genes (Cheng et al., 2014; Rojas et al., 2015, 2019; Aguilar et al., 2016; Sepulveda et al., 2017a; Local et al., 2018). Moreover, MLL3/4 depletion results in transcriptional activation of some MLL3/4-target genes as both Set1a/b-COMPASS and MLL1-COMPASS-like gain access to these genes to mediate the transition from H3K4me1 to H3K4me3 that accompanies transcription (Cheng et al., 2014). Together, these studies imply that different COMPASS and COMPASS-like complexes bind to target sequences in a highly coordinately manner to first maintain a gene silent, but poised for transcription, and subsequently to activate its expression.

Chromatin domains with decreased enrichment of H3K4me3 or H3K27me3 can also be maintained through the activity of lysine demethylases (Dimitrova et al., 2015; Jambhekar et al., 2019). In particular, demethylation of H3K4me3 in mammals is mediated by members of the Jarid1/Kdm5 family (Jarid1a, b, c, and d) (Kooistra and Helin, 2012), which convert H3K4me3 and H3K4me2 to H3K4me1 (Christensen et al., 2007; Iwase et al., 2007; Yamane et al., 2007). Absence of these enzymes can result in enrichment of H3K4me3 at target genes and transcriptional activation (Albert et al., 2013; Rojas et al., 2015). On the other hand, histone demethylases Utx/Kdm6a and Jmjd3/Kdm6b can catalyze the removal of methyl groups from H3K27me3 and therefore counteract the silencing activity of PRC2 (Agger et al., 2007; Hong et al., 2007; Lan et al., 2007; Santa et al., 2007; Xiang et al., 2007).

Methylation at H3K9 (H3K9me1, H3K9me2, and H3K9me3) is strongly associated with the formation of highly compacted and transcriptionally repressed chromatin (heterochromatin) (Saksouk et al., 2015; Nicetto and Zaret, 2019). The methyl-transferases that deposit this modification (“H3K9me writers”) include Suv39H1/Kmt1a, Suv39H2/Kmt1b and Setdb1/ESET/Kmt1e, which can mediate mono-, di-, and tri-methylation. Also, G9a/Ehmt2/Kmt1c and GLP/Ehmt1/Kmt1d, which mediate H3K9me1 and H3K9me2 (Saksouk et al., 2015; Nicetto et al., 2019). All of these enzymes are critical components of several transcription repressive complexes that operate during cell differentiation (Nicetto et al., 2019). The H3K9 methyl-transferase activity is counteracted by H3K9 demethylases, that remove these marks (“H3K9me1/2/3 erasers”) (Janssen et al., 2018). Among them, Lsd1/Kdm1, Jmjd1a/Kdm3a, Jmjd1c/Kdm3c, which can eliminate H3K9me1 or H3K9me2, and Jmjd2a/Kdm4a, Jmjd2b/Kdm4b, Jmjd2c/Kdm4c, and Jmjd2d/Kdm4d, which erase H3K9me1, H3K9me2, and H3K9me3.

Chromatin organization and transcriptional activity is also mediated by ATP-dependent remodelers (Horn and Peterson, 2001; Bakshi et al., 2010; Längst and Manelyte, 2015), which are multi-subunit complexes that include a catalytic core (e.g., Brg1 in the mammalian SWI/SNF and INO80 in the INO80 complex) mediating binding and hydrolysis of ATP (ATPase activity) (Tsukiyama, 2002; Martens and Winston, 2003). These remodelers alter chromatin structure by mobilizing nucleosomes in *cis* or by transferring histone octamers in an ATP-dependent manner. This changes the exposure of regulatory motifs thereby facilitating or preventing recognition by cognate factors (Liu et al., 2011). The contribution of ATP-dependent remodelers during development and differentiation is well-established (Srivastava et al., 2010; Ruijtenberg and van den Heuvel, 2016). Whereas these complexes can be specifically recruited to gene promoters by tissue-specific transcription factors (Armstrong et al., 1998; Serna et al., 2001), several reports also indicate that their targeting is modulated by HPTMs, including histone lysine acetylation and histone arginine methylation (Kanno et al., 2004; Pal et al., 2004). This regulation is due to the presence of bromo- and chromo-domains at subunits of these complexes (e.g., SWI/SNF and CHD3/4) that recognize these modified histone residues (Becker and Workman, 2013).

Genomic DNA can be methylated at cytosines that are followed by guanosines (CpG dinucleotides). The DNA methyl-transferases that mediate this modification belong to a well-conserved family of proteins that include both maintenance (Dnmt1) and *de novo* (Dnmt3a and Dnmt3b) activities (Franchini et al., 2012; Bogdanović and Lister, 2017). Most reports demonstrate that methylated CpG is associated with higher chromatin compaction and reduced transcriptional activity (Franchini et al., 2012; Bogdanović and Lister, 2017). DNA demethylation in mammalian cells involves the conversion from 5-methyl-CpG (5mCpG) to 5-hydroxymethyl-CpG (5hmCpG) by the activity of the Ten Eleven Transformation (Tet) family of dioxygenases (Tahiliani et al., 2009; Ito et al., 2010; Bogdanović and Lister, 2017). Importantly, Tet proteins form regulatory complexes with chromatin remodelers like SWI/SNF as well as with histone methyl-transferases and histone demethylases (Williams et al., 2011; Yildirim et al., 2011; Neri et al., 2013).

Enrichment of H3K9me3/2 at transcriptionally-inactive chromatin can be associated with the presence of 5mCpG. This is due to the ability of the proteins that “write” and “read” these two epigenetic marks to form complexes (Janssen et al., 2018; Nicetto and Zaret, 2019) thereby providing a means for both repressive epigenetic modifications (5mCpG and H3K9me3) to collaborate in generating a condensed chromatin structure that reduces transcription. These findings further indicate that different epigenetic mechanisms leading to chromatin remodeling and transcriptional control can function in a coordinated and complementary manner within eukaryotic cells, allowing an effective regulation of gene expression in response to physiological cues.

## Epigenetic Control of the Expression of Master Regulators of Osteogenesis

**FIGURE 1:**
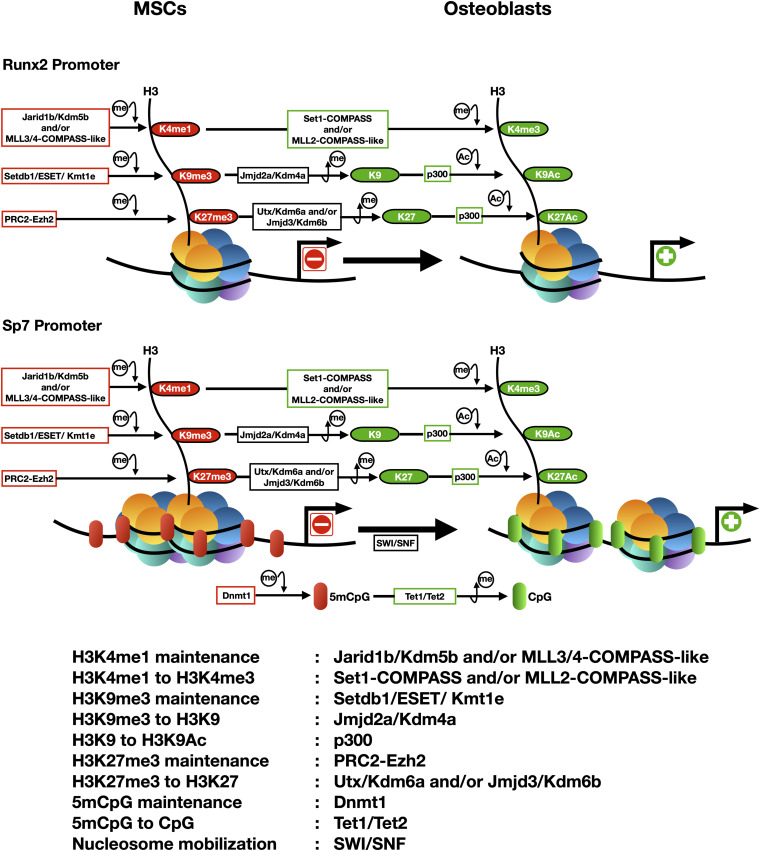
Epigenetic landscapes at the transcriptionally active and repressed Runx2 and Sp7 gene promoters during osteogenic differentiation of mesenchymal stem cells (e.g., CD10 positive hMSCs). The arrowhead indicates the transcriptional start site (TSS). Principal epigenetic features and each enzyme associated are included and summarized below.

Differentiation of mesenchymal stem cells (MSCs) to the osteogenic lineage requires the expression and function of two master transcription factors: Runx2 (Runt Related Transcription Factor 2) and Sp7 (also known as Osterix). These factors bind to and control the expression of numerous downstream target genes that code for proteins that are necessary for establishing the osteoblast phenotype (Zhang, 2010; Sinha and Zhou, 2013; Meyer et al., 2014; Wu et al., 2014). Runx2 and Sp7 are expressed at early embryonic stages of the mesenchyme-osteoblast lineage commitment process and optimal concentrations of both factors in pre-osteogenic cells is essential for proper osteoblast differentiation (Long, 2012). Runx2 binds to and activates the Sp7 promoter (Yoshida et al., 2012), indicating that Runx2 is an upstream transcription factor during osteogenesis, but that this differentiation process requires the expression of both master regulators (Long, 2012). In recent years, several groups have demonstrated that changes in chromatin structure and expression of the Runx2 and Sp7 genes are epigenetically-controlled (Yang et al., 2013, 2015; Tai et al., 2014; Dudakovic et al., 2015; Rojas et al., 2015, 2019; Zhang et al., 2015; Aguilar et al., 2016, 2020; Park-Min, 2016; Zhou et al., 2016; Sepulveda et al., 2017a; Cakouros and Gronthos, 2020; Figure 1).

### Methylation at Histone H3K4 and H3K27 Residues

Active transcription of the Runx2 and Sp7 genes is accompanied by an epigenetic landscape that includes increased H3K4me3 enrichment at both promoter regions (Figure 1). Hence, transcription of these master genes requires binding and activity of COMPASS and COMPASS-like complexes (Shilatifard, 2012). Studies initiated two decades ago first demonstrated that over-expression of Wdr5, a critical subunit of these complexes, accelerates osteoblast and chondrocyte differentiation programs (Gori et al., 2001, 2006; Zhu et al., 2008). Wdr5 binds to the Runx2-P1 promoter in osteoblastic cells (Rojas et al., 2015) where it can mediate transcriptional upregulation. It was recently found that transcription of both Runx2 and Sp7 during osteogenesis requires that Set1-COMPASS and MLL2-COMPASS-like complexes mediate deposition of the H3K4me3 mark at both promoters (Rojas et al., 2015, 2019; Sepulveda et al., 2017b).

It has been determined that an enrichment of H3K4me1 accompanies transcriptional silence at the Runx2 and Sp7 genes in non-osteoblastic and mesenchymal uncommitted cells (Rojas et al., 2015; Aguilar et al., 2016, 2020; Sepulveda et al., 2017a,b). As MLL3/4-COMPASS can mediate maintenance of H3K4me1 at repressed, but poised for expression, genes (Cheng et al., 2014; Rojas et al., 2015, 2019; Aguilar et al., 2016; Sepulveda et al., 2017a; Local et al., 2018), one possibility is that H3K4me1 is deposited at the Runx2 and Sp7 promoters by the MLL3/4 complexes that have been found associated with both genes in MSCs prior to engage osteogenesis (Sepulveda et al., 2017b; Rojas et al., 2019).

Maintenance of chromatin with low H3K4me3 enrichment (and hence elevated H3K4me1) at the Runx2 promoter can be also achieved by binding and activity of the H3K4 demethylase Jarid1b/Kdm5b (Figure 1; Rojas et al., 2015, 2019; Bustos et al., 2017; Sepulveda et al., 2017a), which converts H3K4me3 to H3K4me1 (Christensen et al., 2007; Iwase et al., 2007; Yamane et al., 2007). Targeted depletion of Jarid1b/Kdm5b in mice results in elevated lethality at early post-natal stages, exhibiting these animals cranial dysmorphic parameters and skeletal alterations (Albert et al., 2013). Moreover, silencing of Runx2 expression during myogenic cell differentiation is mediated by Jarid1b/Kdm5b bound to the Runx2 promoter (Rojas et al., 2015, 2019). Accordingly, knockdown of Jarid1b/Kdm5b expression is accompanied by enrichment of H3K4me3 at the Runx2 promoter and active Runx2 transcription in mouse mesenchymal pluripotent cells differentiating to non-osteoblastic lineages (Rojas et al., 2015; Bustos et al., 2017; Sepulveda et al., 2017a).

Findings from several teams revealed that regulating the repressive mark H3K27me3 represents an important step during the differentiation of MSCs toward osteoblasts. It was demonstrated that the activity of the H3K27 demethylases Utx/Kdm6a and Jmjd3/Kdm6b is critical for erasing this mark from the promoter of the Runx2 and Sp7 genes (Figure 1) and hence to induce their expression during osteogenesis (Ye et al., 2012; Hemming et al., 2014; Rojas et al., 2015; Park-Min, 2016; Sepulveda et al., 2017b; Cakouros and Gronthos, 2020; Sen et al., 2020). Additionally, an increased activity of the PRC2 complex that “writes” the H3K27me3 mark, can significantly limit the ability of MSCs to engage osteogenic differentiation (Wei et al., 2010). Moreover, forced expression of Ezh2 in pre-osteoblastic cells results in significant downregulation of osteogenic genes, including Runx2 and its downstream targets (Hemming et al., 2014). Accordingly, knockdown of Ezh2 expression in MSCs and pre-osteoblastic cells results in increased expression of the Runx2 (Rojas et al., 2015, 2019; Hemming et al., 2016) and Sp7 (Sepulveda et al., 2017b) genes.

### Methylation at the Histone H3K9 Residue

The repressive mark H3K9me3 plays an important role during differentiation of MSCs to the osteoblastic lineage (Ye et al., 2012, 2018; Lawson et al., 2013; Tai et al., 2014; Rojas et al., 2015; Zhang et al., 2015; Sepulveda et al., 2017a,b). H3K9me3 is found enriched at the Runx2 and Sp7 promoters in cells that do not express these genes (Figure 1) and binding of proteins that can write and read this mark has been detected in non-osseous cells (Zhang et al., 2015; Aguilar et al., 2016; Sepulveda et al., 2017b). The H3K9 methyl transferase Setdb1/ESET/Kmt1c binds to the Sp7 promoter and maintains a H3K9me3-enriched transcriptionally-silent environment in mouse MSCs and other non-osseous cells (Sepulveda et al., 2017b). Accordingly, during BMP2-induced osteogenic differentiation of MSCs, Setdb1/ESSET/Kmt1c is released from this promoter and replaced by the H3K9me3 demethylase Jmjd2a/Kdm4a, an exchange that is accompanied by decreased enrichment of H3K9me3 and transcription of the Sp7 gene (Sepulveda et al., 2017b). Interestingly, it was also established that a different member of this family of demethylases, Jmjd2b/Kdm2b, plays a critical role during formation of the mouse skeleton (Ye et al., 2012, 2018). However, this demethylase neither directly binds nor decreases the H3K9me3 enrichment at the Sp7 and Runx2 promoters (Ye et al., 2012, 2018).

Although most studies support the role of the H3K27me3 and H3K9me3 marks during repression of both master osteogenic genes, recent analyses indicate that for certain cells, this role may have to be re-evaluated. Thus, analysis of uncommitted human umbilical cord-derived Wharton Jelly MSCs (WJ-MSCs) showed that the Runx2-P1 promoter region does not exhibit significant enrichment of H3K9me3 and H3K27me3 (Bustos et al., 2017; Sepulveda et al., 2017a). Interestingly, both marks are detected at the Sp7 gene promoter in these cells and represent a relevant component of the epigenetic barrier preventing the expression of Sp7 when the cells are induced to differentiate to the osteoblastic lineage (Bustos et al., 2017; Sepulveda et al., 2017a). Hence additional mechanisms are preventing the expression of Runx2-p57 in these uncommitted WJ-MSCs in the absence of H3K27me3 and H3K9me3. One proposed component is the H3K4 demethylase Jarid1b/Kdm5b which was found enriched at this Runx2 P1 promoter concomitant with elevated H3K4me1 (Bustos et al., 2017; Sepulveda et al., 2017a). Knockdown of Jarid1b/Kdm5b expression or selective inhibition of Jarid1b/Kdm5b activity using small molecules, is accompanied by enrichment of H3K4me3 at the Runx2 promoter and Runx2 upregulation in these WJ-MSCs (Bustos et al., 2017; Sepulveda et al., 2017a). Future studies need to carefully address what alternative mechanisms control the expression of Runx2 and Sp7 in the different sources of human MSCs available.

### Histone H3 and H4 Acetylation

Histone lysine acetylation also plays a relevant role during bone formation as it supports increased expression of critical genes for osteoblast differentiation (Schroeder and Westendorf, 2005; McGee-Lawrence and Westendorf, 2011). Chromatin remodeling and transcription of the Runx2 gene is accompanied by acetylation of histones H3 and H4 (Figure 1) in nucleosomes associated with the P1 promoter region (Lee H. W. et al., 2006; Hassan et al., 2007; Cruzat et al., 2009; Gordon et al., 2011). This acetylation is correlated with decreased expression of HDACs (HDAC1, 2, and 3) during BMP2-induced osteoblast differentiation (McGee-Lawrence and Westendorf, 2011). Accordingly, knockdown of HDAC1 and HDAC3 expression facilitates osteogenic differentiation (Maroni et al., 2012) and a number of HDAC inhibitors [Sodium Butyrate, Trichostatin A, Valproic acid, and Suberoyl Anilide Hydroxamic Acid (SAHA)] can promote osteogenesis in models including primary calvarial cells and MSCs (Cho et al., 2005; Schroeder and Westendorf, 2005; Lee H. W. et al., 2006; Haberland et al., 2010; Hu et al., 2012; Dudakovic et al., 2013; Zych et al., 2013). Moreover, the histone acetyl transferase (HAT) p300 plays a major role in maintaining histone acetylation at the Runx2-P1 (Rojas et al., 2015) and Sp7 (Sepulveda et al., 2017b) gene promoters (Figure 1).

### DNA Methylation

DNA methylation (5mCpG) can regulate osteogenic differentiation of MSCs. Addition of the DNA methyl-transferase inhibitor 5-Azacytidine (5-Aza) to human and murine mesenchymal cells enhances their ability to engage osteogenesis by reducing 5mCpG enrichment at regulatory regions of osseous genes (Lee J. Y. et al., 2006; Zhou et al., 2009; Zych et al., 2013; Sepulveda et al., 2017b; Cakouros et al., 2019). In particular, an active Sp7 gene promoter exhibits reduced 5mCpG at its proximal promoter (Figure 1), whereas this region shows increased 5mCpG in non-osseous cells that do not express Sp7 (Lee J. Y. et al., 2006; Sepulveda et al., 2017b). Moreover, during BMP2-induced osteoblast differentiation DNA demethylation of the Sp7 promoter is mediated by a Tet1/Tet2-containing complex which transforms 5mCpG to 5hmCpG (Sepulveda et al., 2017b). This process is tightly coordinated with nucleosome remodeling mediated by SWI/SNF, “erasing” of the H3K9me3 and H3K27me3 repressive marks and “writing” of the H3ac and H3K4me3 active marks (Sepulveda et al., 2017b; Figure 1). A recent report indicates that in mouse bone marrow-derived MSCs (BM-MSCs), Tet1 is pre-bound to osteogenic gene promoters in the absence of Tet2, playing an initial repressor role. This may result from the recruitment of repressor activities, including Sin3A, Hdacs, and PRC2 (Cakouros et al., 2019). Interestingly, Tet1 can also play a key role during transcriptional activation of osteogenic genes. Tet1 recruits Tet2 to mediate 5mCpG demethylation and gene transcription during differentiation of BM-MSCs (Cakouros et al., 2019). Together these results support a model where binding of Tet1 may initially contribute to maintain target osteogenic genes (including Runx2) silent but poised for expression as the MSCs engage osteoblast lineage commitment.

## Concluding Remarks

Controlling lineage commitment in MSCs represents a critical challenge to overcome skeletal deficiencies in patients. Extensive research during the last 20 years has shed light into potential targeting of specific epigenetic mechanisms that control bone-related gene expression. Future studies will need to consider the influence of the mechanical environments at which MSCs are maintained and differentiated *ex vivo*. Recent results demonstrate that the substrate stiffness at which hMSCs are grown in culture can induce rapid and stable changes in chromatin organization and the associated epigenetic landscapes that permanently up- or down-regulate genes (Killaars et al., 2019), hence modifying the ability of these cells to engage osteogenesis. Future research also needs to develop approaches that include safe delivery strategies that limit undesired effects of treatments based on epidrugs on non-target tissues and organs, thereby decreasing potential negative secondary effects in treated patients.

## Author Contributions

MM analyzed the data and wrote most parts of the manuscript. MC wrote parts of the text and prepared the figures. GN analyzed the data, wrote parts of the text and helped preparing the figures. All authors contributed to the article and approved the submitted version.

## Conflict of Interest

The authors declare that the research was conducted in the absence of any commercial or financial relationships that could be construed as a potential conflict of interest.
